# Insulin deficiency is associated with early postpartum T2DM in Indian women with gestational diabetes

**DOI:** 10.3389/fcdhc.2026.1788084

**Published:** 2026-04-28

**Authors:** Puja Chebrolu, Mallika Alexander, Shilpa Naik, Megh Prajapati, Priyanka Raichur, Samhita Bellamkonda, Sanjaykumar Tambe, Vandana Kulkarni, Aditi Kole, Sanghamitra Pati, Myung Hee Lee, Daniel W. Fitzgerald, Todd T. Brown, K. M. Venkat Narayan, Jyoti S. Mathad

**Affiliations:** 1 Weill Cornell Medicine, New York, NY, United States; 2 Byramjee Jeejeebhoy Medical College, Pune, India; 3 Cornell University, Ithaca, NY, United States; 4 Johns Hopkins India Private Limited, Pune, India; 5 University of Pennsylvania, Philadelphia, PA, United States; 6 Clinical Trials Unit, Byramjee Jeejeebhoy Medical College, Pune, India; 7 Indian Council of Medical Research, New Delhi, India; 8 Johns Hopkins University School of Medicine, Baltimore, MD, United States; 9 Emory Global Diabetes Research Center, Woodruff Health Sciences Center and Emory University, Atlanta, GA, United States

**Keywords:** gestational diabetes, India, insulin deficiency, insulin resistance, postpartum diabetes

## Abstract

**Introduction:**

Women with gestational diabetes mellitus (GDM) are at increased risk of type 2 diabetes mellitus (T2DM). Understanding the pathophysiology of postpartum T2DM is crucial for prevention. We conducted a prospective study to characterize insulin mechanics in postpartum T2DM among women with GDM.

**Materials and methods:**

GDM was diagnosed by oral glucose tolerance test (OGTT) or oral glucose challenge test. We administered an OGTT at 6 weeks and 3, 6, and 12 months postpartum. Postpartum T2DM was defined by the American Diabetes Association criteria at any time point. We assessed whether the insulinogenic index (a measure of early-phase insulin secretion) or the Matsuda index (a measure of insulin sensitivity) at 6 weeks postpartum was associated with T2DM at any time point.

**Results:**

Of 100 women [median age 28 years (IQR 25–32), BMI 27.6 kg/m^2^ (IQR 23.8–30.6) at 6 weeks postpartum], 42% (39) developed T2DM by 1 year postpartum. Among those with T2DM, 67% (26) were diagnosed at 6 weeks postpartum. Age, height, and baseline weight were similar in women with and without T2DM. Baseline triglycerides were higher (127.6 vs. 93.1 mg/dL, p=0.02) among women with T2DM. In proportional hazards analysis, a higher insulinogenic index at 6 weeks postpartum was protective against T2DM [aHR 0.24, 95% CI (0.11, 0.51], independent of age, BMI, triglycerides, and insulin use. The Matsuda index was not associated with T2DM (p=0.53).

**Conclusions:**

Over 40% of women with GDM developed T2DM by 12 months postpartum, most within 6 weeks. Insulin secretion was more strongly associated with T2DM than insulin sensitivity. Earlier screening and further study of the role of beta-cell preservation may help reduce T2DM among young Indian women.

## Introduction

Women who develop gestational diabetes mellitus (GDM) are at high risk of progressing to type 2 diabetes mellitus (T2DM) postpartum (1). While studies from the US and Europe suggest that 10% of women with GDM will develop T2DM within 25 years postpartum, women in India may progress more rapidly (2–4). A study from Chennai, India suggests that 90% of Indian women with GDM will develop T2DM within 10 years postpartum (5) while data from Pune, India suggests that progression is even more rapid—27% of women with GDM were diagnosed with T2DM in the first year postpartum (6). Despite these alarming statistics, there are limited data on the contribution of insulin resistance and insulin deficiency to postpartum T2DM among women with a history of GDM.

Insulin deficiency may be a major yet underappreciated contributor to T2DM pathophysiology, particularly among Indian adultsamong (7). In a cross-sectional comparative study, insulin secretion and insulin resistance were three times lower among adults with T2DM in Chennai, India, compared to Pima Indian adults in the United States (who are at high risk for diabetes) (8). A similar pattern of low insulin secretion was observed in a comparison of Indian and Swedish women with GDM (9). The relative insulin deficiency in Asian Indians compared to other ethnic groups could be due to early life factors, such as malnutrition, that limit pancreatic and adipose tissue growth and alter epigenetic expression of insulin production-related genes (10). A predominant insulin deficiency may also explain why lifestyle interventions are less effective among Asian Indians compared to US adults (11, 12). Few studies have longitudinally followed Asian Indian women with GDM to determine risk factors for postpartum T2DM, especially in low-income populations. We aimed to determine the association of insulin secretion and resistance with postpartum diabetes among women with a history of GDM. We hypothesized that insulin deficiency is more strongly associated with postpartum T2DM than insulin resistance in women with GDM.

## Methods

### Study design

We conducted a prospective study at Sassoon General Hospitals (SGH) in Pune, India, between 2021 and 2024.

### Study population and eligibility criteria

SGH is a tertiary care government hospital that primarily serves low-income populations and provides free medical care. Pregnant and postpartum women ≥18 years of age were enrolled from the antenatal care clinic (ANC) in the third trimester or from the postnatal ward at SGH. All women attending the ANC or postnatal ward during the study period were screened for eligibility. Women were included if they had a chart diagnosis of GDM, primarily made by International Association of Diabetes in Pregnancy Study Group (IADPSG) or Diabetes in Pregnancy Study Group India (DIPSI) criteria, or if their glucose values met IADPSG criteria even if a GDM diagnosis was not listed in the chart (13, 14). Women who were not planning to stay in Pune postpartum, were unwilling or unable to consent, or those who did not have documented GDM screening results were not enrolled. Women with known diabetes prior to pregnancy or diagnosed in the first trimester of pregnancy were also excluded.

### Ethical procedures

Informed consent was obtained in Marathi or Hindi, depending on the participant’s native language. We obtained ethical approval from the ethical review committee at Byramjee Jeejeebhoy Government Medical College in India and the Institutional Review Boards at Weill Cornell Medicine and Johns Hopkins University School of Medicine in the United States.

### Study procedures

The following data were obtained at 6 weeks and 3, 6, and 12 months postpartum: demographic and anthropometric measurements (weight, height, mid-upper arm circumference, and waist circumference), blood pressure (measured using a manual sphygmomanometer), body composition (measured using the InBody 770 bioimpedance analyzer (15)), and plasma lipids (total cholesterol, LDL-c, HDL-c, and triglycerides). A fasting 75-g oral glucose tolerance test (OGTT), with glucose and insulin measured at fasting, 30 min, and 120 min, was performed at each postpartum time point. Participants were instructed to fast for at least 8 h before each OGTT.

**Insulin ELISA:** Approximately 1 mL of peripheral whole blood was collected in a 4-mL red-top (plain), clot activator Vacutainer tube (Cat #369032, Manufacturer: Becton, Dickson and Company (BD), New Jersey, USA). The sample was allowed to clot at room temperature for 30 min and then centrifuged at 2,400 rpm for 10 min. The separated serum was aliquoted and stored at −80°C until batch testing. Serum insulin concentrations were measured using the Invitrogen™ Human Insulin ELISA Kit (Catalog # KAQ1251) according to the manufacturer’s instructions (16). Briefly, 50 µL of serum sample, standard, and blank were added to the designated microplate wells, followed by the addition of anti-insulin horseradish peroxidase conjugate to each well. The plate was incubated at room temperature for 30 min. After incubation, the wells were aspirated and washed three times with the wash buffer. Subsequently, 100 µL of chromogen solution was added to each well within 15 min of completing the washing step, and the plate was incubated at room temperature in the dark for 15 min. The stop solution was added to each well, resulting in a color change from blue to yellow. Optical density was measured at 450 nm within 1 h using a microplate reader. A standard curve was generated using a four-parameter logistic regression model, and insulin concentrations in the samples were calculated accordingly. This assay was tested for cross-reactivity with proinsulin and found to be non-reactive (16).

Data on GDM screening, treatment, pregnancy complications, and delivery outcomes were obtained via chart review.

### Variables, outcomes, and statistical analyses

The primary outcome was new diagnosis of T2DM, defined as meeting the American Diabetes Association (ADA) criteria for T2DM at any postpartum time point: fasting glucose ≥126 mg/dL or 2-h glucose ≥200 mg/dL. Prediabetes, as defined by ADA criteria, was a secondary outcome.

Baseline characteristics were described for all women. Continuous variables were summarized by median and interquartile range (IQR), and categorical variables were summarized by frequency and percentage. BMI was categorized using the cutoffs for Asian people: underweight (≤18 kg/m^2^), normal weight (18–23 kg/m^2^), or overweight (≥23 kg/m^2^) (17). High waist circumference was defined as ≥80 cm per World Health Organization criteria (18, 19). Delivery outcomes were abstracted from delivery records. Infant birth-weight percentile was calculated using weight and gestational age at delivery using the INTERGROWTH-21 calculator (20).

The Δ symbol indicates change in values. Insulin production was calculated using multiple formulas to assess first- and second-phase insulin secretions and to adjust for insulin resistance: (1) insulinogenic index (IGI) (Δinsulin 0–30 min/Δglucose 0–30 min), (2) oral disposition index (oDI) (IGI*1/fasting insulin), and (3) first-phase insulin production (Δinsulin 0–30 min). Because their distribution was skewed, the insulinogenic index and oDI were log-transformed for Cox regression analysis. Insulin resistance was calculated using the Matsuda index (10,000/√[(fasting glucose × fasting insulin)(mean glucose × mean insulin)]. Δinsulin production and Δinsulin resistance measures from 6 weeks to 12 months postpartum were compared between women with and without T2DM using the Mann–Whitney U test.

T2DM-free survival over 12 months was described using a Kaplan–Meier curve, and cumulative T2DM incidence was estimated. A Cox proportional hazards model was used to examine the association of insulin production and insulin resistance at 6 weeks with the detection of T2DM by 12 months. Data were censored at the first loss to follow-up. Participants were included until 12 months postpartum or until diagnosis of T2DM, whichever occurred first. Known risk factors for T2DM, including age, BMI, triglycerides, and insulin use during pregnancy, were included in the model. With 100 samples, assuming a 40% event rate, the minimum detectable hazard ratio (HR) at a power of 80% was 2.4. The proportional hazards assumption was assessed visually and formally using Schoenfeld residuals. We found no evidence that the proportional hazards assumption was violated (p=0.97). Sensitivity analyses with more and less parsimonious models were conducted (see the Supplementary data). To evaluate selection bias related to loss to follow-up, the characteristics of women who were lost to follow-up after 6 weeks postpartum were compared to those who attended the 12-month follow-up visit (**Supplementary Table 1**).

## Results

### Screening and enrollment

**Figure 1:**
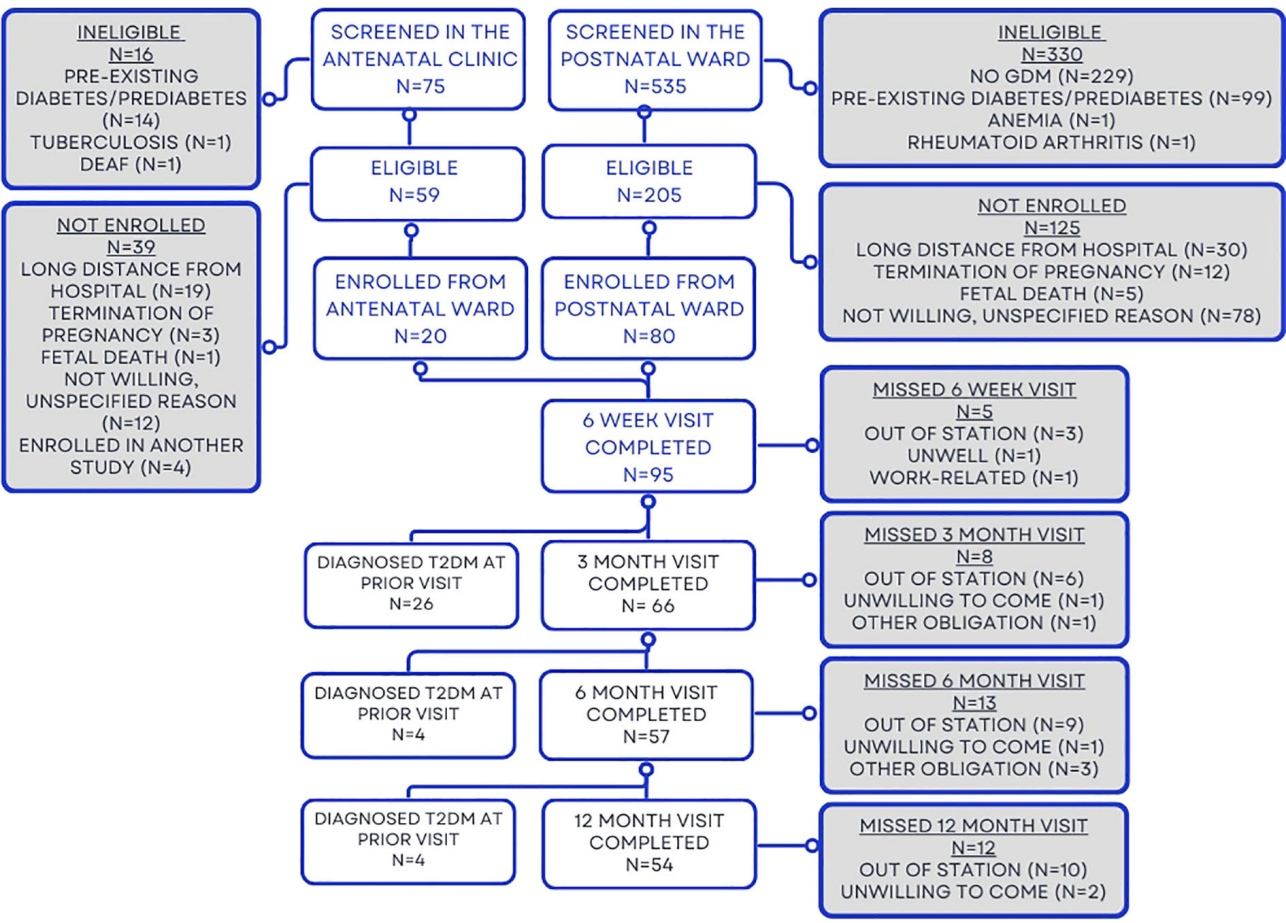
Participant numbers at each study visit.

A total of 535 women were approached from the postnatal ward, with 80 enrolled. Seventy-five women were approached from the antenatal clinic, with 20 enrolled, for a total of 100 women enrolled. Reasons for ineligibility and non-enrollment are provided in **Figure 1**.

### Study population and cumulative T2DM and prediabetes

A total of 100 women attended at least one study visit (**Figure 1**). The median age of the cohort was 28 years (IQR 25–32), the median gestational age at GDM screening was 28.3 weeks (IQR 16.7–33.3), and the median BMI at 6 weeks postpartum was 27.6 kg/m^2^ (IQR 23.8–30.6). Six percent (n=6) had GDM in a prior pregnancy (**Table 1**). Thirty-one women (31%) had a preterm birth.

**Table 1:** Baseline^a^ characteristics and anthropometrics among participants with and without T2DM.

Variable	Total N (%) (N = 100)	Newly diagnosed T2DM by 12 months N (%) (N = 39)	No T2DM by 12 months N (%) (N = 61)	P value
Median age, years (IQR)	28 (25–32)	28 (25–32)	28 (25–31)	0.41
Gestational age at first screening (weeks)	28.3 (16.7–33.3)	28.2 (17.3–33)	28.7 (15–34.1)	0.94
Lower socioeconomic status^#^	48 (48.0%)	15 (38.5%)	33 (54.0%)	0.25
Family history of DM	23 (23%)	12 (30.8%)	11 (18.0%)	0.14
History of GDM in a pregnancy prior to this one	6 (6%)	5 (12.8%)	1 (1.6%)	0.02
GDM medications				
Orals (metformin, sulfonylureas, DPP-4 inhibitors)	48 (48%)	19 (48.7%)	29 (47.5%)	<0.01
Insulin	21 (21%)	17 (43.6%)	4 (6.6%)	
Pregnancy and delivery outcomes				
Pre-eclampsia	18 (18%)	11 (28.2%)	7 (11.5%)	0.03
C-section	68 (68%)	28 (71.8%)	40 (65.6%)	0.52
Preterm birth	31 (31%)	19 (48.7%)	12 (19.7%)	<0.01
LGA (>90th percentile birth weight)	10 (10%)	3 (7.7%)	7 (11.5%)	0.54
Baseline anthropometrics and laboratory results [median (IQR)]				
Height (cm)	152.7 (147.8, 157)	152 (148, 157)	152.9 (147, 157)	0.85
Weight (kg)	62.4 (53.3, 75.3)	62.7 (51.3, 75.2)	62.0 (54.4, 76.1)	0.66
BMI (kg/m^2^)	27.6 (23.8, 30.6)	28.0 (23.6, 30.4)	27.4 (23.9, 31.8)	0.71
Waist circumference (cm)	95 (87.5, 102)	95 (88.5, 102)	94.3 (86, 102)	0.71
LDL (mg/dL)	107.0 (86.8, 123.1)	111.3 (95.1, 129.8)	102.6 (84.4, 119.6)	0.15
HDL (mg/dL)	45.3 (39.0, 51.9)	42.4 (34.7, 49.9)	47.2 (40.8, 52.5)	0.08
Triglycerides (mg/dL)	102.0 (74.1, 140.8)	127.6 (85.2, 171.1)	93.1 (74.1, 123)	0.02
Insulin calculations [median (IQR)]				
IGI at 6 weeks	0.43 (0.18, 0.94)	0.16 (0.06, 0.33)	0.67 (0.40, 1.21)	<0.01
oDI at 6 weeks	0.04 (0.02, 0.08)	0.02 (0.01, 0.03)	0.05 (0.04, 0.12)	<0.01
Matsuda index at 6 weeks	4.68 (3.42, 6.63)	4.12 (2.81, 5.16)	5.09 (3.80, 7.39)	0.02
First phase insulin secretion at 6 weeks (μIU/mL)	26.31 (10.8, 42.23)	9.61 (4.50, 23.38)	31.19 (23.50, 62.70)	<0.01
Change in IGI from 6 weeks to 12 months^b^	0.03 (-0.14, 0.30)	0.09 (-0.01, 0.15)	0.02 (-0.62, 0.48)	0.88
Change in oDI from 6 weeks to 12 months^b^	0.01 (0.001, 0.07)	-0.01 (-0.04, 0.003)	0.05 (0.002, 0.08)	0.01
Change in Matsuda index from 6 weeks to 12 months^b^	1.80 (-0.95, 5.20)	0.21 (-1.57, 3.06)	2.80 (0.36, 5.46)	0.44

^#^Kuppuswamy socioeconomic status scale; *log-transformed; IQR, interquartile range; T2DM, type 2 diabetes mellitus; GDM, gestational diabetes mellitus; LGA, large for gestational age; SGA, small for gestational age; LBW, low birth weight; BMI, body mass index; LDL, low-density lipoprotein; HDL, high-density lipoprotein; IGI, insulinogenic index; and oDI, oral disposition index.;.

^a^At 6 weeks postpartum and ^b^data available for 21 participants.

**Figure 2:**
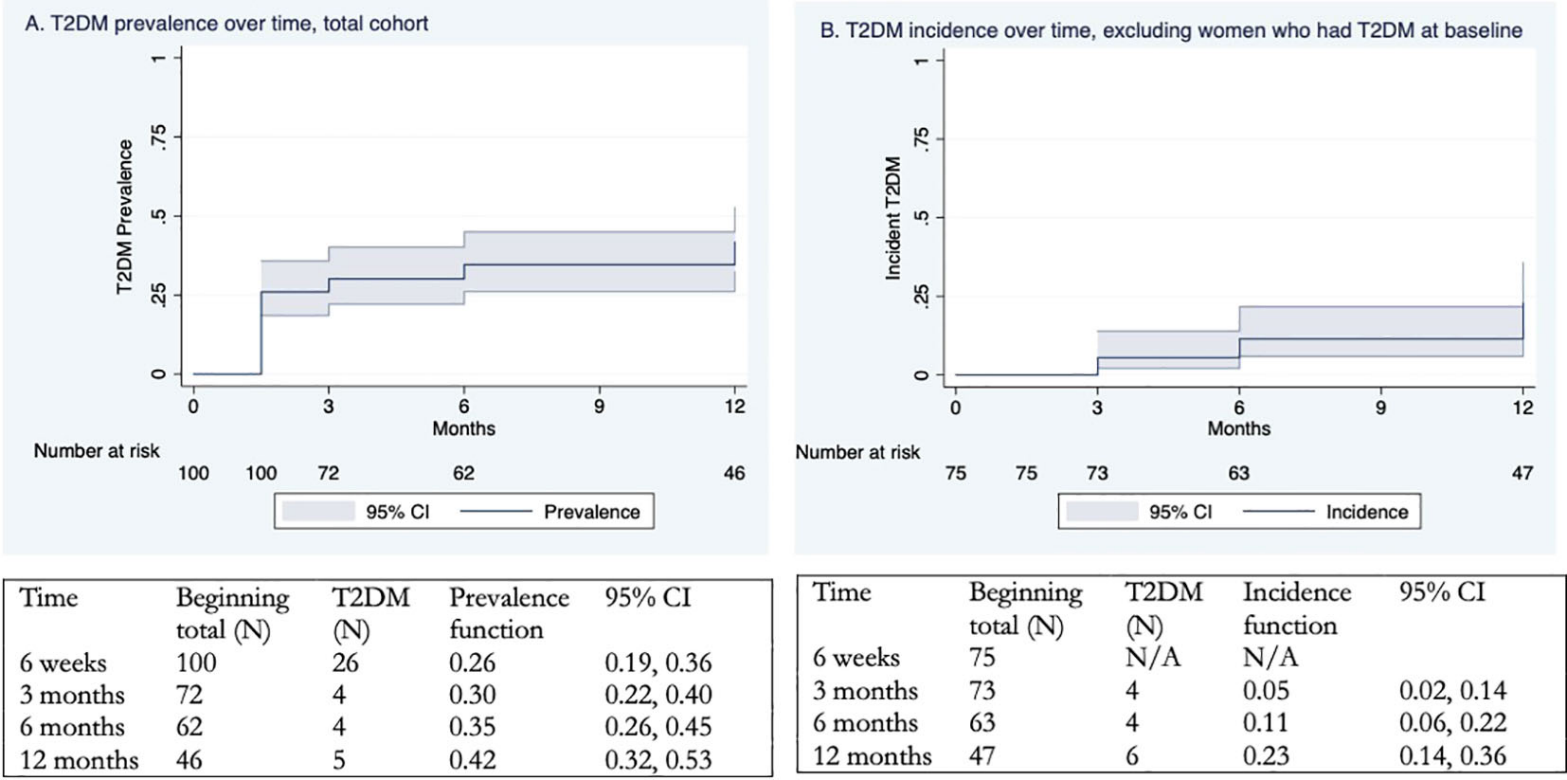
**(a)** Newly diagnosed T2DM over 12 months postpartum using Kaplan–Meier estimates. **(b)** Newly diagnosed T2DM over 12 months postpartum using Kaplan–Meier estimates, excluding women who had T2DM at baseline (6 weeks postpartum).

**Figure 3:**
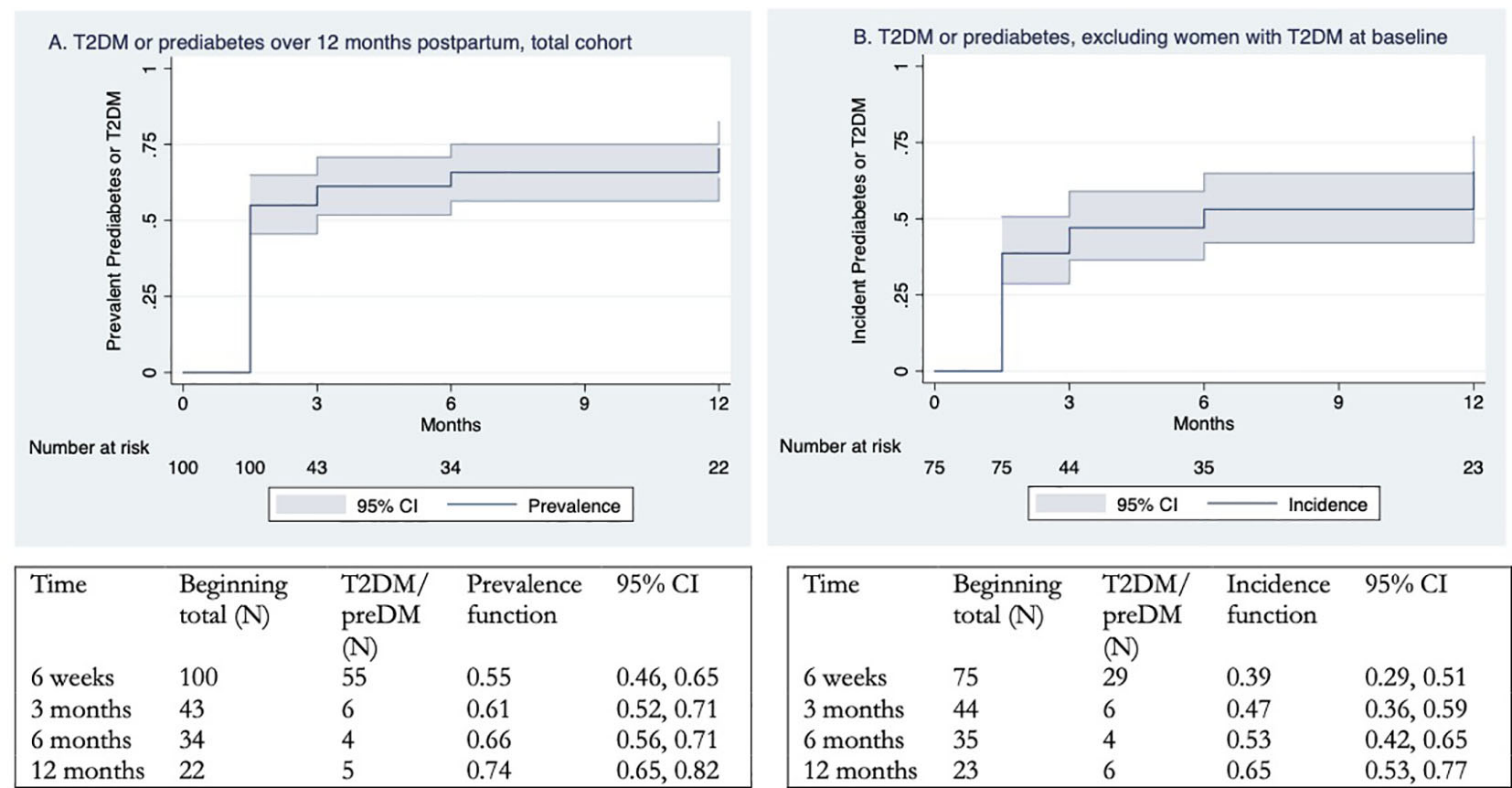
**(a)** Newly diagnosed T2DM or prediabetes over 12 months postpartum using Kaplan–Meier estimates. **(b)** Newly diagnosed T2DM or prediabetes over 12 months postpartum using Kaplan–Meier estimates, excluding women who had T2DM at baseline (6 weeks postpartum).

Cumulative T2DM by 12 months postpartum was 42% (n=39, 95% CI 32%–53%) (**Figure 2a**). Even when excluding women with T2DM at 6 weeks postpartum (to represent a scenario where they all had pre-gestational T2DM), the cumulative T2DM remained 23% (N = 14) (**Figure 2b**). Cumulative T2DM or prediabetes by 12 months postpartum was 74% (n=70, 95% CI 65%–82%) (**Figure 3a**), or 65% (N = 45) when women with T2DM at 6 weeks were excluded (**Figure 3b**). Of the women who had T2DM by 12 months, 66.7% (n=26) had T2DM by 6 weeks postpartum, and 20.5% (n=8) had prediabetes at 6 weeks postpartum (**Figure 3**).

### Comparison of risk factors among women with and without T2DM by 12 months postpartum

There was no difference in the following parameters at 6 weeks postpartum between women with and without T2DM by 12 months: age (28 vs. 28 years), height (152 vs. 152.9 cm), weight (62.7 vs. 62.0 kg), waist circumference (95 vs. 94.3 cm), or HDL-c (42.4 vs. 47.2 mg/dL). Women with T2DM by 12 months postpartum had a greater prevalence of GDM in a prior pregnancy (12.8% vs. 1.6%), use of insulin during pregnancy (43.6% vs. 6.6%), and higher 6-week postpartum triglycerides (127.6 vs. 93.1 mg/dL) and total cholesterol (184.0 vs. 167.8 mg/dL). There was no difference in the change in weight or waist circumference from 6 weeks to 12 months postpartum between women with and without T2DM by 12 months (**Table 1**).

### Insulin parameters among women with and without T2DM by 12 months postpartum

**Figure 4:**
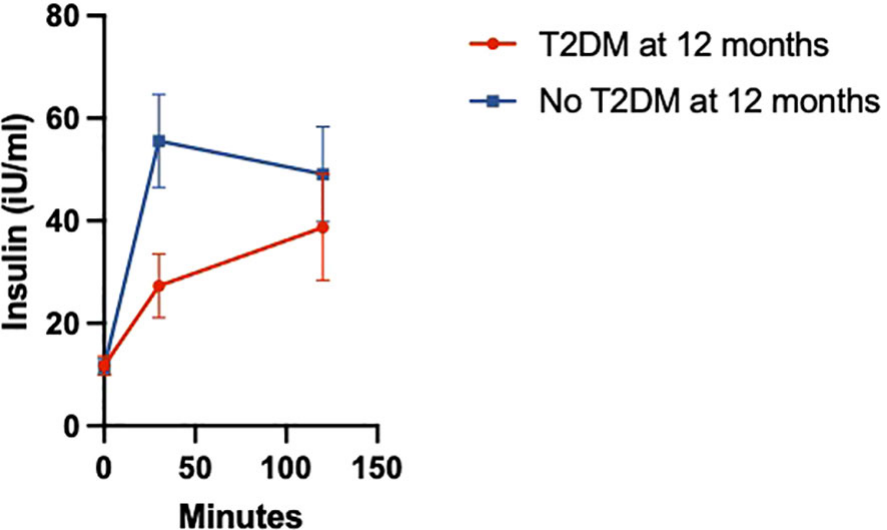
Median insulin secretion during OGTT at 6 weeks in women with and without T2DM by 12 months.

Insulin levels during OGTT at 6 weeks postpartum exhibited a slower rise and later peak in women who had T2DM by 12 months postpartum compared to women without T2DM (**Figure 4**). Women with T2DM also had significantly lower fasting insulin at 6 weeks postpartum (9.6 vs. 31.0 iU/mL), IGI (0.16 vs. 0.67, p<0.01), and oDI (0.02 vs. 0.05, p<0.01) than women without T2DM. Women with T2DM also had a lower Matsuda index (4.1 vs. 5.1, p=0.02) at 6 weeks postpartum than women without T2DM. Between 6 weeks and 12 months postpartum, the ΔoDI was smaller in women with T2DM compared to women without T2DM by 12 months (-0.01 vs. 0.05, p=0.01). However, the ΔIGI (0.09 vs. 0.02, p=0.88) and ΔMatsuda index (0.21 vs. 2.80, p=0.44) from 6 weeks to 12 months postpartum were similar regardless of T2DM status (**Table 1**).

On multivariate Cox proportional hazards analysis, a higher insulinogenic index was associated with a lower hazard of T2DM (aHR 0.20, 95% CI 0.09, 0.46, p<0.01) independent of age, BMI, triglycerides, or insulin use during pregnancy. A higher oDI was associated with a lower hazard of T2DM (aHR 0.22, 95% CI 0.10, 0.49, p<0.01). The Matsuda index did not show similar associations with T2DM (p=0.53) (**Table 2**). Sensitivity analysis results with a more parsimonious model excluding triglycerides are reported in **Supplementary Table 3**.

**Table 2:** Multivariable Cox proportional hazards analysis of the association between insulin parameters and T2DM.

Variable	Model 1^a^ aHR (95% CI)	P-value	Model 2^b^ aHR (95% CI)	P-value	Model 3^c^ aHR (95% CI)	P-value
Age (years)	1.00 (0.93, 1.08)	0.94	1.01 (0.94, 1.09)	0.76	1.02 (0.95, 1.11)	0.58
BMI^#^ (kg/m^2^)	0.99 (0.92, 1.06)	0.77	0.98 (0.91, 1.05)	0.55	0.98 (0.91, 1.05)	0.64
Triglycerides^#^ (mg/dL)	1.00 (1.00, 1.01)	0.36	1.00 (0.99, 1.01)	0.86	1.00 (1.00, 1.01)	0.25
Insulin use during pregnancy	6.72 (1.36, 33.1)	0.02	9.54 (1.93, 47.1)	<0.01	13.1 (2.78, 61.8)	<0.01
Insulinogenic index*^#^	0.20 (0.09, 0.46)	<0.01				
Oral disposition index*^#^			0.22 (0.10, 0.49)	<0.01		
Matsuda index^#^					0.96 (0.82, 1.12)	0.58

^#^At 6 weeks postpartum; ^*^log transformed; ^a^includes insulinogenic index; ^b^includes oral disposition index; ^c^includes Matsuda index.

## Discussion

In our cohort, more than 40% of women with a history of GDM were found to have T2DM by 12 months postpartum, with most (67%) exhibiting abnormal results (T2DM or prediabetes) by 6 weeks postpartum. Insulin resistance—often the target for T2DM prevention (11, 21)—was less strongly associated with T2DM in this population compared to decreased insulin production. Our results highlight the importance of low insulin production as a factor in postpartum T2DM in South Asian women. The early postpartum diagnosis suggests that glucose abnormalities may be present in young women before pregnancy. Preconception and early postpartum screening for prediabetes and T2DM in young Indian women may identify high-risk women early and allow for interventions to prevent long-term adverse outcomes.

The cumulative prevalence of T2DM in our cohort of women from a government hospital in Pune is higher than that reported in other studies of Asian women. From a private hospital in Pune, a study of 220 women with higher- income reported a 27% prevalence of T2DM within 12 months postpartum, while a study of 19 centers across Bangladesh, Sri Lanka, and India reported a 17.1% incidence by 1.8 years postpartum (6, 22). Postpartum abnormalities in glucose tolerance were highly prevalent in our cohort, which had a comparatively lower median age and lower socioeconomic status. One potential contributor to this high prevalence is undernutrition. Although T2DM is classically considered a condition of overnutrition, there is increasing evidence that undernutrition, particularly early-life undernutrition, independently predisposes adults to T2DM. One hypothesis is that organ development may be restricted due to limited nutrient supply, or epigenetic changes from deficient nutrients such as vitamin B12 may occur (23–25). What we detected as a new diagnosis of T2DM may, in fact, be a worsening of glucose intolerance that existed prior to pregnancy but was undetected. Despite a median age of only 28 years, 6% of women had GDM in a prior pregnancy.

Traditional risk factors such as older age, higher BMI, or large-for-gestational-age babies were not correlated with T2DM in this study. These “traditional” risk factors are associated with greater insulin resistance (26). However, in the South Asian population, reduced insulin production may play a more important role in T2DM pathophysiology than insulin resistance. South Asians tend to have the subtype of T2DM now called severe insulin-deficient diabetes (SIDD), which is currently poorly understood (27). Multiple studies in non-pregnant populations show that insulin production in South Asians is much lower than in US or European adults (8, 27, 28). We now show significant insulin deficiency in a cohort of young postpartum women who had GDM during pregnancy. Causes of this pathophysiology may include genetic and epigenetic changes that affect insulin production, malnutrition, low birth weight, and exposure to pollutants, all of which can affect beta-cell mass and therefore insulin production (29–33).

Lower insulin production or beta-cell function was significantly associated with T2DM in our cohort. While higher baseline insulin production (as assessed by IGI and oDI) was protective against T2DM, increased insulin resistance (as measured by the Matsuda index) was not significantly associated with T2DM on regression analysis. We also noted elevated triglycerides in women who developed T2DM. Elevated triglycerides are usually associated with central obesity and insulin resistance. In our population, however, we propose that high triglycerides are an indirect marker of inadequate insulin, as insulin is necessary to clear triglycerides from the blood (34). In pregnancy, GDM pathophysiology is classically attributed to excess insulin resistance, where a level of insulin resistance that exceeds the ability of the pancreatic beta cells to compensate with insulin production leads to GDM (35). However, recent data suggest that insulin production is actually independent of insulin resistance during pregnancy (36). In women with GDM, therefore, beta-cell dysfunction may occur independently of insulin resistance and persist postpartum. A few other cohorts, particularly those that enrolled Asian women, support our findings of a greater role for beta cell decline than insulin resistance in postpartum T2DM (37, 38). One cohort of 186 Chinese women with a history of GDM found that 6-week insulinogenic index and HOMA-B (a measure of insulin production) predicted T2DM better than HOMA-IR, a measure of insulin resistance (38). This was confirmed in a more recent Chinese study among 1,263 women with a history of GDM which found that, among non-obese women, HOMA-B had a greater contribution to postpartum diabetes than HOMA-IR (39). This difference in pathophysiology is important because landmark studies on postpartum T2DM prevention recommend interventions to improve insulin sensitivity, including metformin, thiazolidinediones, carbohydrate reduction, and exercise (11, 21, 40). Our findings may help explain why these interventions have been less effective in Asian populations. Understanding T2DM pathophysiology is essential to inform prevention efforts and provide effective treatment (11, 12, 41).

Our study had multiple strengths and limitations. We prospectively enrolled women with GDM and conducted multiple follow-up oral glucose tolerance tests within the first year. Our retention rate was relatively high, with 54 of 66 eligible women (i.e., those not yet diagnosed with T2DM by the 12-month visit) completing OGTT at 12 months (82%). Ninety-three percent of them had at least two follow-up visits. We compared women with at least two follow-up visits to those without and found baseline characteristics and risk factors to be similar between groups (**Supplementary Table 1**). Our retention was better than other large studies with similar term postpartum follow-ups (42). Our study focused on low-income and postpartum women, both of whom are underrepresented in T2DM research. Because T2DM is growing rapidly among low- and middle-income countries, it is especially important to understand the spectrum of pathophysiology in these populations. One limitation of our study is that we enrolled women with a pre-existing diagnosis of GDM rather than conducting universal screening of pregnant women. This may have resulted in the enrollment of women with more severe GDM and limited our ability to include data on treatment during pregnancy. Women were also diagnosed by different methods (IADPSG and DIPSI), though comparison of diagnoses by these methods did not show any significant differences (**Supplementary Table 2**). We also did not have a non-GDM comparator cohort in this study. We now have an ongoing study at the same site where all pregnant women are being prospectively screened for GDM, with insulin testing planned for women with and without GDM. GDM is not a condition of pregnancy alone; metabolic changes may start well before pregnancy (43, 44). Future studies should screen women for abnormal glucose tolerance prior to pregnancy. Future research should also examine the impact of preserving and improving beta cell function on the development of postpartum T2DM.

## Conclusion

Postpartum glucose abnormalities were detected soon after pregnancy in 74% of our cohort of low-income Indian women with GDM, suggesting the need for early screening, including prior to pregnancy. We found that insulin deficiency was more strongly associated with T2DM than insulin resistance in this population. While current prevention and treatment measures focus on insulin resistance, further longitudinal studies on the impact of preserving and improving beta-cell function may be required to effectively prevent T2DM in certain populations.

## Data Availability

The original contributions presented in the study are included in the article/**Supplementary Material**. Further inquiries can be directed to the corresponding author.
