# Supplementary material for: Insulin deficiency is associated with early postpartum T2DM in Indian women with gestational diabetes
Source: Front Clin Diabetes Healthc. 2026 Apr 28;7:1788084. doi: 10.3389/fcdhc.2026.1788084 (PMC13162042; doi:10.3389/fcdhc.2026.1788084)
Supplement: Supplementary file 1 [file Table1.docx]

Supplemental Table 1. Comparison of women who did and did not attend at least 2 follow-up visits

|  | Attended at least 2 visits (N=93) | Did not attend at least 2 visits (N=7) | P value |
| --- | --- | --- | --- |
| Median age, years (IQR) | 28 (25-31) | 32 (27-32) | 0.16 |
| Family history of diabetes (%) | 21 (22.6%) | 2 (28.6%) | 0.72 |
| Median BMI, kg/m2 (IQR) | 27.4 (23.4-30.6) | 28.9 (28.3-29.9) | 0.23 |
| Median triglycerides, mg/dl (IQR) | 102.7 (74.1-138.0) | 86.8 (84.8-171.1) | 0.93 |
| Diagnostic criteria |  |  | 0.25 |
| DIPSI | 12 (12.9%) | 2 (28.6%) |  |
| IADPSG | 5 (71.4%) | 81 (87.1%) |  |
| Medication use during pregnancy (%) |  |  | 0.57 |
| Orals (metformin, sulfonylureas, DPP-4 inhibitors) | 46 (49.5%) | 2 (28.6%) |  |
| Insulin | 19 (20.4%) | 2 (28.6%) |  |

Supplemental Table 2. Comprehensive table of baseline characteristics among women with and without T2DM by 12 months

|  | Total  N (%)  (N=100) | T2DM by 12 months  N (%)  (N=39) | No T2DM by 12 months  N (%)  (N=61) | P value |
| --- | --- | --- | --- | --- |
| Socioeconomic status^#^ |  |  |  |  |
| Lower <5 | 5 (5.0%) | 3 (7.7%) | 2 (3.2%) | 0.25 |
| Upper lower 5-10 | 43 (43%) | 12 (30.8%) | 31 (50.8%) |  |
| Lower middle 11-15 | 46 (46%) | 19 (53.8%) | 25 (50.0%) |  |
| Upper middle 16-25 | 6 (6%) | 3 (7.7%) | 3 (4.9%) |  |
| Diagnostic method |  |  |  |  |
| IADPSG | 86 (86%) | 34 (87.2%) | 52 (85.2%) | 0.79 |
| DIPSI | 14 (14%) | 5 (12.8%) | 9 (14.8%) |  |
| Breastfeeding at 12 months |  |  |  |  |
| Exclusive | 36 (53.7%) | 12 (50%) | 24 (55.8%) | 0.27 |
| Mixed | 18 (26.9%) | 9 (37.5%) | 9 (37.5%) |  |
| Formula | 13 (19.4%) | 3 (12.5%) | 10 (23.3%) |  |
|  |  |  |  |  |
| **Pregnancy and delivery outcomes** | | | | |
| SGA (<10^th^ percentile birthweight) | 25 (25%) | 8 (20.5%) | 17 (27.9%) | 0.41 |
| LBW (<2500g) | 31 (31%) | 11 (28.2%) | 20 (32.8%) | 0.63 |
|  |  |  |  |  |
| **Anthropometrics and laboratory results (baseline)** | | | | |
| Median Body fat%, % (IQR) | 42.1 (38.5, 46.8) | 41.8 (36.7, 45.5) | 43.2 (38.9, 47.7) | 0.18 |
| Median visceral fat area, cm^2^ (IQR) | 146.2 (114.9, 184.5) | 140 (102.4 183.8) | 150.9 (118.6, 186.8) | 0.30 |
| Median total cholesterol, mg/dl (IQR) | 173.6 (151.4 200.8) | 184.0 (162.1, 215.4) | 167.8 (145.6, 195.4) | 0.02 |
|  |  |  |  |  |
| Median fasting glucose, mg/dl (IQR) | 94.9 (87.2-102.8) | 117.7 (100.6-204.9) | 91.9 (85.4-95.7) | <0.01 |
| Median 30 min glucose, mg/dl (IQR) | 154.1 (137.5-174.2) | 194.6 (151.1-278.2) | 149.5 (136.7-163.6) | 0.01 |
| Median 2-hour glucose, mg/dl (IQR) | 136.6 (106.2-175.1) | 246.3 (181.8-363.9) | 126.2 (105.6-148.7) | <0.01 |
|  |  |  |  |  |
| **Change in parameters from 6 weeks to 12 months** | | | | |
| Median weight, kg (IQR) | 0 (-3.4, 2.3) | -0.2 (-3.2, 2.8) | 0 (-3.8, 2.3) | 0.60 |
| Median waist circumference, cm (IQR) | -2 (-6, 5) | -1.45 (-5.5, 6) | -2 (-7, 5) | 0.65 |
| Median visceral fat, cm^2^ (IQR) | -10.8 (-27.8, 10.8) | -7.2 (-13.3, 11.8) | -11.4 (-29.2, 10.8) | 0.53 |
| Median body fat %, %, (IQR) | -1.7 (-5.8, 2) | -1.1 (-2.7, 1.5) | -1.9 (-6.5, 2) | 0.51 |

Supplemental Table 3. Multivariable Cox proportional hazards analysis of the association between insulin parameters and T2DM, most parsimonious model

|  | Model 1^a^  aHR (95% CI) | p-value | Model 2^b^  aHR (95% CI) | p-value | Model 3^c^  aHR (95% CI) | p-value |
| --- | --- | --- | --- | --- | --- | --- |
|  |  |  |  |  |  |  |
| Age, years | 0.99 (0.92, 1.06) | 0.81 | 1.01 (0.94, 1.08) | 0.79 | 1.02 (0.94, 1.10) | 0.63 |
| BMI^#^, kg/m^2^ | 0.99 (1.01, 1.07) | 0.79 | 0.98 (0.92, 1.04) | 0.55 | 0.99 (0.92, 1.06) | 0.64 |
| Insulin use during pregnancy | 7.86 (1.67, 37.0) | 0.01 | 9.9 (2.17, 45.7) | <0.01 | 15.3 (3.36, 69.9) | <0.01 |
| Insulinogenic index*^#^ | 0.19 (0.08, 0.41) | <0.01 |  |  |  |  |
| Oral disposition index*^#^ |  |  | 0.19 (0.09, 0.40) | <0.01 |  |  |
| Matsuda index^#^ |  |  |  |  | 0.93 (0.80, 1.07) | 0.31 |

^#^at 6 weeks postpartum; ^*^log transformed

Supplemental Table 4. Multivariable Cox proportional hazards analysis of the association between insulin parameters and T2DM, excluding women who had T2DM at 6 weeks postpartum (baseline)

|  | Model 1^a^  aHR (95% CI) | p-value | Model 2^b^  aHR (95% CI) | p-value | Model 3^c^  aHR (95% CI) | p-value |
| --- | --- | --- | --- | --- | --- | --- |
|  |  |  |  |  |  |  |
| Age, years | 1.00 (0.85, 1.17) | 0.97 | 1.00 (0.86, 1.16) | 0.99 | 1.02 (0.87, 1.20) | 0.79 |
| BMI^#^, kg/m^2^ | 1.00 (0.86, 1.16) | 0.998 | 0.99 (0.85, 1.14) | 0.86 | 1.01 (0.87, 1.17) | 0.89 |
| Triglycerides^#^, mg/dl | 1.01 (0.99, 1.03) | 0.58 | 1.00 (0.98, 1.03) | 0.69 | 1.01 (0.99, 1.03) | 0.27 |
| Insulin use during pregnancy | 7.03 (0.35, 143) | 0.20 | 11.8 (0.53, 262.2) | 0.12 | 7.65 (0.41, 142.3) | 0.17 |
| Insulinogenic index*^#^ | 0.15 (0.01, 1.69) | 0.12 |  |  |  |  |
| Oral disposition index*^#^ |  |  | 0.09 (0.003, 2.27) | 0.14 |  |  |
| Matsuda index^#^ |  |  |  |  | 1.18 (0.86, 1.61) | 0.31 |

^#^at 6 weeks postpartum; ^*^log transformed

Supplemental Table 5. Multivariable Cox proportional hazards analysis of the association between insulin parameters and T2DM, including method of GDM diagnosis

|  | Model 1^a^  aHR (95% CI) | p-value | Model 2^b^  aHR (95% CI) | p-value | Model 3^c^  aHR (95% CI) | p-value |
| --- | --- | --- | --- | --- | --- | --- |
|  |  |  |  |  |  |  |
| Age, years | 1.00 (0.93, 1.08) | 0.94 | 1.01 (0.94, 1.09) | 0.75 | 1.02 (0.95, 1.11) | 0.79 |
| BMI^#^, kg/m^2^ | 0.99 (0.92, 1.06) | 0.78 | 0.98 (0.91, 1.05) | 0.58 | 0.98 (0.91, 1.05) | 0.55 |
| Triglycerides^#^, mg/dl | 1.00 (0.99, 1.01) | 0.58 | 1.00 (0.99, 1.01) | 0.88 | 1.00 (1.00, 1.01) | 0.24 |
| Insulin use during pregnancy | 6.74 (1.36, 33.5) | 0.02 | 9.68 (1.94, 48.4) | 0.01 | 13.1 (2.79, 61.9) | <0.01 |
| Diagnosis by IADPSG | 1.03 (0.38, 2.75) | 0.96 | 1.07 (0.40, 2.90) | 0.89 | 1.19 (0.44, 3.26) | 0.73 |
| Insulinogenic index*^#^ | 0.20 (0.09, 0.46) | <0.01 |  |  |  |  |
| Oral disposition index*^#^ |  |  | 0.19 (0.08, 0.44) | <0.01 |  |  |
| Matsuda index^#^ |  |  |  |  | 0.96 (0.82, 1.13) | 0.61 |

^#^at 6 weeks postpartum; ^*^log transformed

Supplemental Figure 1. Visual representation of proportional hazards in the cohort
